# Hospital Admissions from Nursing Homes: Rates and Reasons

**DOI:** 10.1155/2011/247623

**Published:** 2011-04-10

**Authors:** Kjell Krüger, Kristian Jansen, Anders Grimsmo, Geir Egil Eide, Jonn Terje Geitung

**Affiliations:** ^1^Løvåsen Teaching Nursing Home, Municipality of Bergen, N-5145 Fyllingsdalen, Norway; ^2^Department of Public Health and General Practice, Norwegian University of Science and Technology, N-7491 Trondheim, Norway; ^3^Centre for Clinical Research, Haukeland University Hospital, N-5021 Bergen, Norway; ^4^Research Group on Lifestyle Epidemiology, Department of Public Health and Primary Health Care, University of Bergen, N-5021 Bergen, Norway; ^5^Department of Radiology, Haraldsplass Hospital, N-5021 Bergen, Norway

## Abstract

Hospital admissions from nursing homes have not previously been investigated in Norway. During 12 months all hospital admissions (acute and elective) from 32 nursing homes in Bergen were recorded via the Norwegian ambulance register. The principal diagnosis made during the stay, length of stay, and the ward were sourced from the hospital's data register and data were merged. Altogether 1,311 hospital admissions were recorded during the 12 months. Admissions from nursing homes made up 6.1% of the total number of admissions to medical wards, while for surgical wards they made up 3.8%. Infections, fractures, cardiovascular and gastri-related diagnoses represented the most frequent admission diagnoses. Infections accounted for 25.0% of admissions, including 51.0% pneumonias. Of all the admissions, fractures were the cause in 10.2%. Of all fractures, hip fractures represented 71.7. The admission rate increased as the proportion of short-term beds increased, and at nursing homes with short-term beds, admissions increased with increasing physician coverage. Potential reductions in hospitalizations for infections from nursing homes may play a role to reduce pressure on medical departments as may fracture prevention. Solely increasing physician coverage in nursing homes will probably not reduce the number of hospitalizations.

## 1. Background

 It is expected that nursing homes will play an important role in health care delivery in the years ahead. The population is growing older, and the patients admitted to hospitals are being discharged earlier. A Norwegian white paper states that reform is needed to the collaboration between primary care and hospitals [1]. The growth in costs and utilization of hospitals is not sustainable. Among several proposals, the white paper points to accomplishments involving early discharges from hospitals to nursing homes, which offer structured rehabilitation programmes. Evaluations have shown a reduction in mortality, readmission, and later need for home care for elderly patients [2]. Palliative units in nursing homes have also been a success [3].

On the other hand, little research has been done on admissions from nursing home to hospital. There are no studies in this field from Norway [4]. Internationally, a correlation has been found between the lack of documented decisions on the level of treatment and the increase in admission rate [5]. Clear documentation in logs and records concerning hospital admissions and heart-lung do-not-resuscitate decisions can prevent unnecessary admissions. Acknowledged routines currently in place to treat terminal patients (Liverpool Care Pathway) may be important to make these decisions easier in nursing homes [6]. We also know that information gaps commonly occur when elderly patients are transferred from a nursing home or seniors' residence to the hospital [7].

Norway has 4.6 million inhabitants, 55 public hospitals, 41,052 nursing home beds, and 1,796 beds in old people's homes, 96.8% of nursing home beds are in single rooms, and 43,3% of all deaths (total 41,342) are in nursing institutions [8]. The healthcare system is split in first- and second-line services. The second line contains the 55 hospitals and specialist services (included private specialists). Second line is administered and financed directly from the state. First-line services are administered and financed by the municipalities (*N* = 431). First line covers general practice (GP) services, mother and child care, home care, and nursing homes (*N* = 900).

The study was approved by the Norwegian Social Science Data Services.

This study investigates the incidence of hospitalizations from nursing homes, the major diagnostic reasons, what burden these hospitalizations represent for the main hospital departments, and what impact manpower and short- to long-term bed ratio have.

## 2. Materials and Method

**Figure 1:**
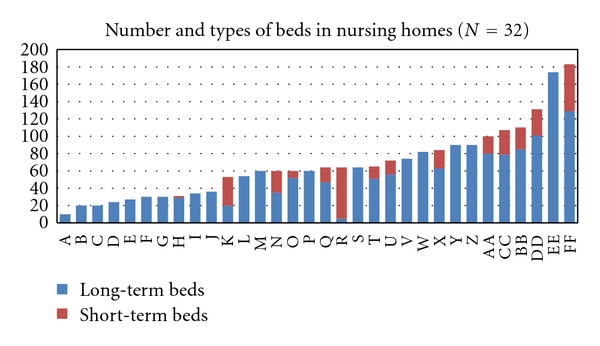
Size and proportion of long-term and short-term beds in all 32 nursing homes in the city of Bergen (250 000 inhabitants), Norway. The red part indicates short-term beds (3-4 weeks stays) and the blue part long-term beds (permanent stays).

In 2007, the Norwegian city of Bergen had approximately 250,000 inhabitants and in total 32 nursing homes (2,300 beds, including 300 short-term beds), see Figure 1. Long-term beds are for permanent residents, mostly until their death. Short-term beds are mainly for 3-4 weeks rehabilitation stays after hospitalization. In nursing homes in Bergen, about 50% of physician services are performed by doctors in permanent positions and the rest by GPs in part-time positions.

During the period from March 2006 to March 2007 (12 months), all hospital inpatient admissions from nursing homes in the Municipality of Bergen to the primary and referral hospital were recorded (the two only hospitals in Bergen). This was done by searching the ambulance service's register of transports from nursing home addresses to the two hospitals. These patient transports were then compared with the hospitals' case history registers to find ward, diagnoses (ICD-10), and length of stay. The ambulance register was the only complete source to find fairly complete figures on hospitalizations from nursing homes for this retrospective study. To search more information about each patient from the hospital records was beyond the scope of the study. Calculations are based on the principal diagnosis. Information about physician manpower, number of total beds and number of short-term beds was gathered from the county health administration.

Two hospitals are delivering hospital services to the city and surrounding municipalities. Bergen population represents 62.5% of the total population served by the hospitals (population 400 000). 

There is only one, public, ambulance service in Bergen. Close to 100 percent of admissions from nursing homes to the hospitals are made by means of ambulance. All transports, patient name, transport addresses, and key medical observations are registered in a database.

Linear regression analysis was used to relate admission rates for the nursing homes to the proportion of short-term beds and the proportion of explained variation in admission rates expressed by the determination coefficient (*R* ^2^). For analyzing data, we used Excel and JMP 8.

## 3. Results

Altogether, 1,311 hospital admissions were recorded during the 12 months. We found a primary diagnosis in 1185 of the cases. The number of admissions was 800 (61.0%) to medical wards (ex pulmonary ward), 385 (29.4%) to surgical wards, 58 (4.4%) to the pulmonary ward, 39 (3.0%) to the neurological ward, and 29 to other wards (2.2%). 

**Figure 2:**
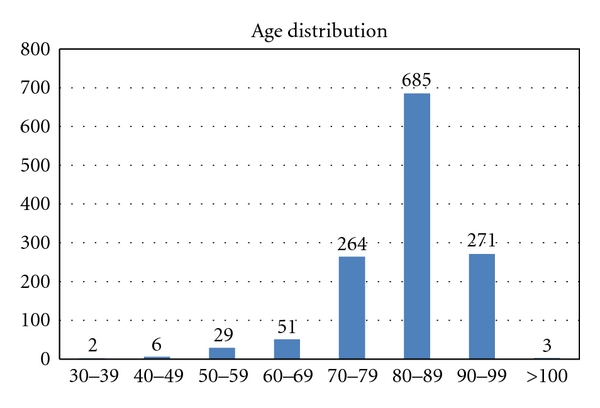
Age distribution of patients admitted to hospital from nursing homes in the city of Bergen, Norway, during the period from March 2006 to March 2007 (*N* = 1311).

**Figure 3:**
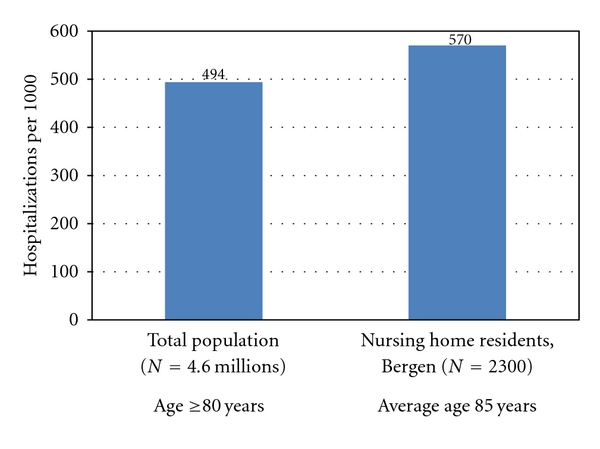
Hospitalization rates (per 1000 inhabitants/residents) for total Norway (*N* = 4.6 millions, age ≥80 years) and among nursing home patients (*N* = 2300, long- and short-term, average age appr. 85 years), 2007, in Bergen, Norway. (Source: Statistics Norway).

The age distribution is shown in Figure 2. 959 (73.2%) patients were at least 80 years of age. Total number of age-specific hospital bed days compared to hospital bed days among nursing home patients is presented in Table 1. Age specific hospitalization rates for total Norway compared to rates among nursing home patients is presented in Figure 3. The diagnoses from hospital stays are presented in Table 2 for the medical and surgical wards. Infections, fractures, and gastrointestinal and cardiac diagnoses stood out as the most frequent reasons for admissions. 

The average length of stay was 4.3 days. There was no significant difference between the average length of stay on medical and surgical wards. A total of 497 (38.0%) of the admissions had a duration of only one inpatient day.

**Figure 4:**
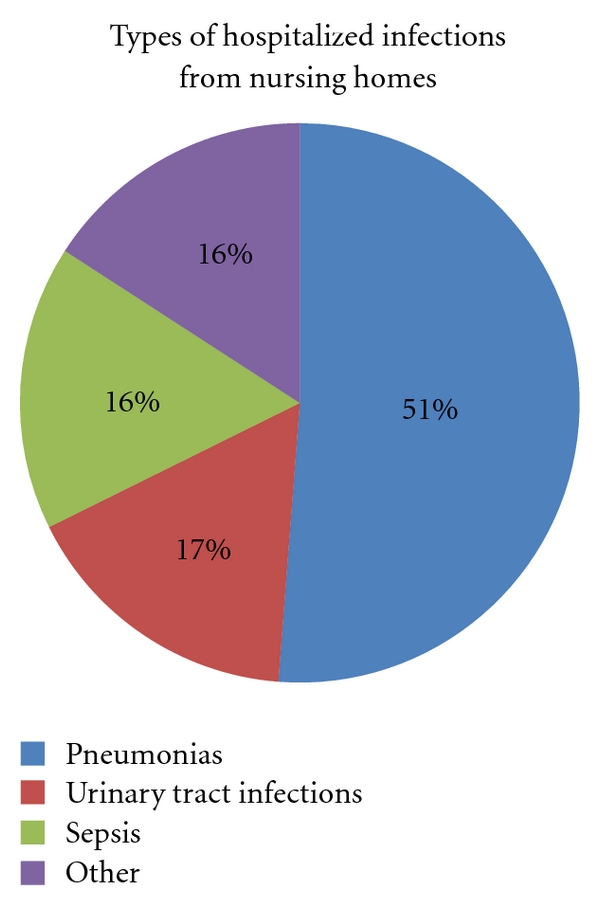
Types of infections on admission from nursing home to hospital in Bergen, Norway, during the period from March 2006 to March 2007 (*N* = 328).

Of all the admissions (1,311), infection diagnoses represented 328 (25.0%). Pneumonias and suspected pneumonias represented 51.0% of infections and 12.8% of all admissions, Figure 4. The incidence of hospitalizations caused by infection was 138/1,000 nursing home beds per year.

**Figure 5:**
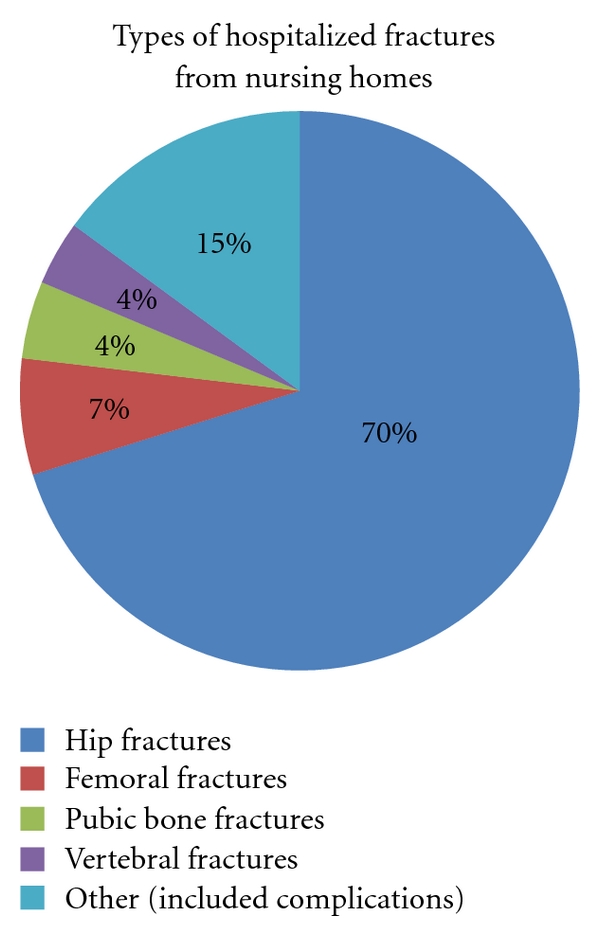
Types of fractures on admission from nursing home to hospital in Bergen, Norway, during the period from March 2006 to March 2007 (*N* = 134).

Fractures were the second most frequent cause counting 134 (10.2%) admissions. Institutions with high fracture rate could have low infection rate and vice versa. The types of fracture are as described in Figure 5. Hip fractures represented 94 (71.7%) of fractures and were the commonest. After excluding the smallest institutions (less than 30 beds) due to probable patient differences, we found a variation in fracture incidence from 0 to 16/100 patient years, the average being 6.4. Only one institution had no fractures. The incidence of fractures treated in hospital among the total population was 5.6/100 patient years during the 12 months under review.

The total number of admissions to medical wards in the two actual hospitals is approximately 21,000 a year, while to surgical wards it is 16,000 admissions [9]. Bergen city represents 62.5% of the total hospital responsibilities. The 800 admissions from nursing homes to the medical wards thus made up 3.8% (6.1% assuming the hospitalization rates from other municipalities had a similar rate) of the total number of admissions. This represented 3440 (5508) bed days calculated on the basis of the average length of stay. For surgical wards the admissions counted for 2.4% (3.8%) and 1656 (2614) bed-days.

**Figure 6:**
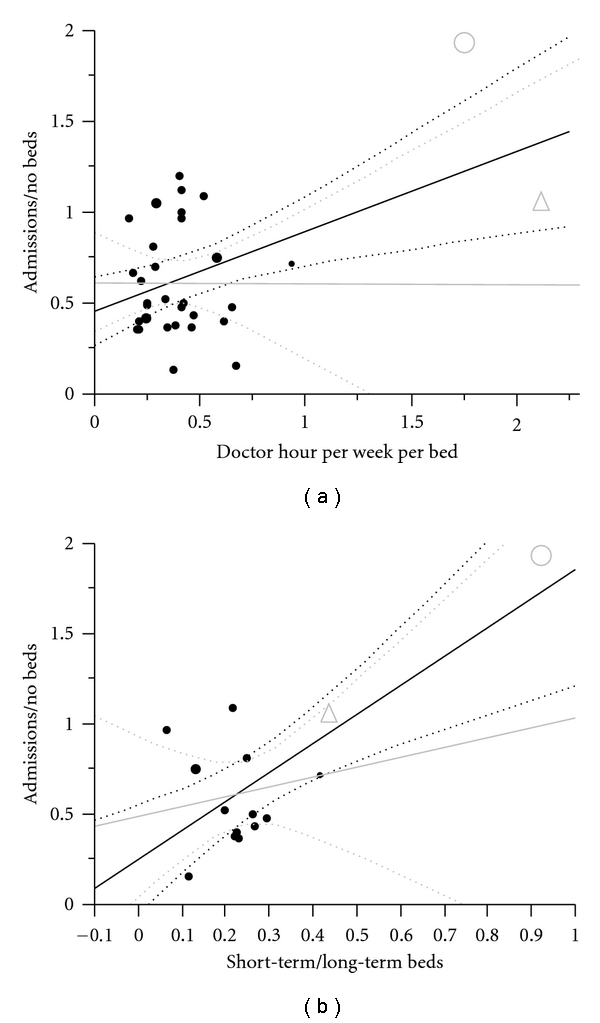
(a) Admissions correlated to physician hours per bed per week (*N* = 32, *R* ^2^ = 0.24, *P* = .0041, *y* = 0.4396*x* + 0.4549), grey line with two institutions with high number of short-term beds and extra staffing excluded. (b) Admissions correlated to short-term/long-term beds. Institutions with zero short-term beds excluded from analysis. Grey line with one institution with almost only short-term beds excluded. (*N* = 15, *R* ^2^ = 0.55/0.04, *P* = .0016/0.51, *y* = 1.6074*x* + 0.2488 for grey line). Dotted lines are confidence curves for regression lines. The grey circle represents institution 32 (92% short-term beds), and the grey triangle represents institution 31 (43% short-term beds).

Number of beds in the nursing homes ranged from 20 to 189. 17 institutions had only long-term beds. The percentage of short-term beds among the 15 mixed homes (long- and short-term beds) ranged from 7% to 92%. Physician manpower ranged from 0.16 to 2.12 hours per bed per week. Linear regression analysis showed a significantly higher admission rate for nursing homes with a high proportion of short-term beds than those with a low proportion (*R* ^2^ = 0.55, *P* = .0016) and also a tendency to higher admission rates as a result of increased physician manpower, see Figure 6. Average physician coverage was 0.48 hours per bed per week, which significantly positively correlated with the percentage of institutional short-term beds.

## 4. Discussion

The incidence of hospitalizations from nursing homes was 570 per 1000 nursing home beds per year. Hospitalizations (age specific) from nursing homes were less than from the general population. Admissions from nursing homes made up 6.1% of the total number of admissions to medical wards while for surgical wards they made up 3.8%. Infections, fractures, cardiovascular and gastri-related diagnoses represented the most frequent admission diagnoses. Infections accounted for 25.0% of admissions, including 51.0% pneumonias. Of all the admissions, fractures were the cause in 10.2%. Of all fractures, hip fractures represented 71.7%. The admission rate increased as the proportion of short-term beds increased, and at nursing homes with short-term beds, admissions increased with increasing physician coverage.

## 5. Limitations of the Study

The role of nursing homes in the delivery of social and health care services differs between, as well as within, countries. Nursing homes in many countries are managed as a part of social care. In Norway nursing homes are regulated as a health care service. These differences may influence the health issue landscape and the composition of staff. Thus, comparisons and generalizations based on our findings should be done with care. 

We estimated that close to 100% of the admissions from nursing homes take place using the ambulance service and therefore considered the basic material reliable. However, it contains no information about the degree of general debilitation or the degree of dementia. This is a weakness. It is difficult to decide whether or not the admissions were unnecessary. The material is too superficial for that. An indication of comorbidity is given by numbers of secondary diagnosis, which range from 0 to 9, averaging 2.9. Our project had access only to anonymous data and could not count readmissions, therefore. This strengthens the material from a patient and data security perspective but calls for further research.

## 6. Reasons for Admissions to Hospital

All have the same right to proper medical treatment in hospitals independent of age. For some old and fragile patients in nursing homes, however, being hospitalized may represent a burden [4]. Both from a patient and society perspective, it may be worth examining ways to reduce hospitalizations for some nursing home patients. 

It has previously been shown that infections are the most common causes of hospital admissions from nursing homes [10–12]. This is confirmed by our results. Most frequent infections in nursing homes have been urinary tract infections (28–41%), respiratory tract infections (25–32%), and skin/soft tissue infections (17–19%) [10, 13–16]. Among hospital admissions we found that urinary tract infections account for 16.5% of infections and pneumonias for 51.2%. One measure that may reduce infection admissions is to ensure that current knowledge about vaccination against influenza and pneumococcal infection is put into practice. Modern nursing homes with small wards may reduce the infection rate of contagious infections, but we found no studies where the impact of these measures on hospitalization rates from nursing home residents has been tested. To reduce hospitalizations due to infections, it is important to secure qualified staff and necessary equipment in nursing homes. Intravenous drug and fluid treatment is needed. Mobile X-ray units can serve several municipalities/nursing homes [17].

Two studies showed that hip fractures had an incidence rate of 3.1% per year among nursing home residents with an average age of 85 [18, 19]. We found a yearly rate of 4.0% but with considerable differences among nursing homes. With regard to measures to prevent fractures, we know that the potential benefit of hip protectors in reducing hip fractures in nursing home residents requires further confirmation [20, 21]. So far Vitamin D supplement seems to give some fracture protection [22]. The great variation in admissions for hip fractures among the institutions, including institutions of comparable size and manpower, should be examined in more detail. This may provide more knowledge about fracture prevention and differences in hospitalization practice. More differentiation in the use of psychoactive drugs for patients at risk of falling could prove valuable like patients with/without the ability to walk unaided.

## 7. The Influence of Manpower

Varying results exist as to the impact the number of nurses has on the frequency of hospital admissions [13, 23, 24]. A retrospective study of 6,623 nursing home patients found that increasing the proportion of nurses cuts the number of short-term patient readmissions, but there was no difference for long-term patients [25]. This seems logical as more manpower makes more advanced treatment possible. 

We found a correlation in the number of admissions from increased physician manpower, with the exception of admissions due to fractures. The need for hospitalization for fractures is probably obvious for all types of health personnel, while many other diagnoses are dependent on diagnostics done by a physician. For example, a study found an increase in the incidence of infections associated with increased physician coverage [24]. An increase in physician manpower in nursing homes might thus have two differentiated effects and be dependent on the composition of diagnoses and the level of diagnostics at the outset. The admission rate will increase for problems better suited to hospitalization and decrease for problems best treated by physicians in the nursing home. Our finding, then, that a higher level of physician manpower was associated with an increased number of admissions may indicate general understaffing of physicians in nursing homes. That increasing proportions of short-term beds leads to increased number of admissions correlates to our expectations. Short-term patients are often still in active treatment relationships with hospital departments and thus probably more frequent readmissions.

55 peer-reviewed articles on interventions that can potentially reduce hospitalizations from formal long-term care settings show the strongest potential for increasing skilled staffing, especially through physician assistants and nurse practitioners [26].

## 8. Conclusions

Monitoring diagnoses and admission rates to hospitals from nursing homes can give a sound basis for evaluating different aspects of running nursing homes. To record “nursing home patient” in the hospital electronic medical record at admission would enlighten research. Optimal treatment of pneumonias in nursing homes may play a role to reduce pressure on medical departments. Solely increasing physician coverage in nursing homes will probably not reduce the number of hospitalizations.

##  Conflict of Interests

The authors declare no conflict of interests.

## Figures and Tables

**Table 1:** Total number of age-specific hospital bed days (all departments and all diagnoses) in the Western Health Region of Norway as compared to hospital bed days among nursing home patients.

Age groups	Bed days in hospital per 1000
0–15 years	401.90
16–49 years	383.33
50–66 years	898.16
67–79 years	2427.20
80 years and above	4322.56
All ages	816.83
Nursing home patients within hospitals' responsibility regions.	3531.30

(Source: Statistics Norway).

**Table 2:** The generic groups for admissions to medical and surgical wards from nursing homes in the city of Bergen, Norway, from March 2006 to March 2007 (*N* = 1185 with diagnosis).

	Medical ward	Surgical ward	Total	As % of admissions (*N* = 1185)
Fractures	9	125	134	11.31
Gastric	91	42	133	11.22
Cardiac	121	2	123	10.38
Infections	267	34	301	25.40
Pulmonary	59	0	59	4.98
Nephrological	23	10	33	2.78
Neurological	69	1	70	5.91
Tumour/cancer	21	27	48	4.05
Urinary tracts	3	66	69	5.82
Other	138	78	215	18.22
Total	801	385	1185	100
